# Anthropometric Parameters, Physical Activity, Physical Fitness, and Executive Functions among Primary School Children

**DOI:** 10.3390/ijerph19053045

**Published:** 2022-03-05

**Authors:** Falonn Contreras-Osorio, Iris Paola Guzmán-Guzmán, Enrique Cerda-Vega, Luis Chirosa-Ríos, Rodrigo Ramírez-Campillo, Christian Campos-Jara

**Affiliations:** 1 Department of Physical Education and Sports, Faculty of Sport Sciences, University of Granada, 18011 Granada, Spain; falonn.contreras@unab.cl (F.C.-O.); lchirosa@ugr.es (L.C.-R.); 2 Exercise and Rehabilitation Sciences Laboratory, Faculty of Rehabilitation Sciences, Universidad Andres Bello, Santiago de Chile 7591538, Chile; 3 Faculty of Chemical-Biological Sciences, Universidad Autónoma de Guerrero, Chilpancingo de los Bravo 39087, Mexico; pao_nkiller@yahoo.com.mx; 4 Pedagogy in Physical Education and Health Career, Department of Health Science, Faculty of Medicine, Pontificia Universidad Católica de Chile, Santiago de Chile 7820436, Chile; ecerdav@uc.cl; 5 Exercise and Rehabilitation Sciences Laboratory, School of Physical Therapy, Faculty of Rehabilitation Sciences, Universidad Andres Bello, Santiago de Chile 7591538, Chile; rodrigo.ramirez@unab.cl

**Keywords:** executive function, anthropometry, physical fitness, human physical conditioning, muscle strength, musculoskeletal and neural physiological phenomena, physical education and training

## Abstract

Physical activity during childhood and adolescence favors brain development and cognitive functioning, particularly the executive functions. This study aimed to assess potential associations between anthropometric parameters, physical activity, physical fitness, and executive functions among elementary school children returning to school after the COVID-19 lockdown in Chile. School-age male and female participants (*n* = 90; age, 10–12 years) participated in the study. To determine the association between variables, a multivariable linear regression analysis was performed. Higher fat-related anthropometric indexes were associated with lower working memory, cognitive flexibility, planning, and attention (r = −0.55 to −0.22; *p* = 0.031 to <0.001). In contrast, higher physical activity levels, better sprint performance, higher lower-body muscular power, and greater upper-body muscular strength were associated with better working memory, cognitive flexibility, inhibition, planning, and/or attention (r = 0.19 to −0.54; *p* = 0.04 to <0.001). Current results consistently suggest the need for adequate levels of physical activity, physical fitness, and anthropometric parameters among the school-age population to promote healthy and adequate executive functions.

## 1. Introduction

Physical activity during childhood and adolescence favors brain development and cognitive functioning [1,2,3,4], particularly the executive functions [5,6,7,8]. In children and adolescents, moderate- and vigorous-intensity physical activity is positively related to working memory [9,10], reaction time [11], executive attention [12], cognitive flexibility, [13] and planning [14]. Moreover, better physical fitness in the school-age population is associated with better planning, working memory, cognitive flexibility, and inhibition [6,15,16,17]. Further, healthier body mass and body mass index are associated with improved general cognitive performance and executive functions in the school-age population [18,19].

Coronavirus disease-19 (COVID-19) triggered the implementation of confinement measures, terminated in-person activities in schools, and gradually restricted free movement to reduce the spread of the virus. There is a large body of information, still under development, describing the consequences of these strategies on children’s lifestyles as well as their mental and physical health [20,21,22,23,24]. Specifically in Chile, all schools were closed on 16 March 2020. As a consequence, children’s educational experiences underwent major changes in terms of reduced access to the school, where much of their physical and sporting activity takes place [25,26], leading to an active discussion about when the children could return to school in person, which was only partially realized during 2021 [27,28].

Confinement measures and restrictions on free movement and changes in children’s physical activity, physical fitness, and cognitive performance, especially in terms of executive functions, which enable the regulation of thoughts and actions while performing goal-directed behavior, have been reported [29]. In this sense, Chambonnière et al. [21] evidenced that physical fitness (i.e., standing long jump performance, medicine ball throwing performance, shuttle run test, maximal aerobic speed) and cognitive performance (i.e., executive function in the Trail Making Test A and B) showed lower performance after the period from February 2020 to January 2021, when the restrictions limiting social interactions in France were lifted. Other authors agree that a decrease in the level of physical activity and sports practice affects children’s physical condition as a consequence of social isolation, especially due to the interruption of the functioning of schools (and their corresponding physical education classes) and sport clubs [20,22,23,24,30,31,32]. On the other hand, Lavigne-Cerván et al. [33] studied the influence of confinement on anxiety, sleep routines, and executive functioning in a sample of children and adolescents (6 to 18 years) using an online questionnaire adapted for parents and demonstrated that anxiety is the variable that best accounts for low executive functioning scores. In Chile, a significant impact of pandemic restrictions on children’s movement behavior, mainly attributable to a decrease in total physical activity and increased screen time, has also been demonstrated [34]. Bustos et al. [35] showed that, one year after the onset of the pandemic, only 19% of children aged 5 to 12 years were engaged in physical activity and/or sports for at least 60 min daily, showing a marked tendency toward physical inactivity and a sedentary lifestyle [36]. 

In Chile, school closures during the most critical pandemic months, beginning from 15 March 2020, and unhealthy lifestyle changes were associated with weight gain in school children [19,21,22,23]. This effect was observed in addition to that described during the summer recess months [24,25] of December, January, and February. Increased body weight in children, in addition to being associated with an increased risk of being overweight or obese, having diabetes, and having metabolic syndrome [26,27,28], is associated with lower cognitive performance, especially with regard to executive functions [29,30]. Supporting this association, Laurent et al. [37] described that a higher body mass index (BMI) was associated with a lower cortical thickness, measured via T1-weighted structural magnetic resonance imaging in a sample of 3190 children aged 9 and 10 years; this association was the strongest for the prefrontal cortex. In this study, the mean prefrontal cortex thickness modulated the association between BMI and working memory. Ronan et al. [38] reported similar results, as they identified that increased BMI was associated with lower scores on a composite measure of executive function among school children aged 9 to 11 years, and they also observed a negative association between executive function and cortical thickness in brain regions involved in decision-making, response inhibition, working memory, and cognitive flexibility.

Based on the background provided by previous research and given the scarcity of studies describing the relationship between anthropometric parameters, physical activity, physical fitness status, and executive functions among primary school children in the context of the COVID-19 pandemic, the purpose of this study was to analyze the possible associations between physical activity, physical fitness, anthropometric parameters, and executive functions among primary school children when they return to the classroom in person after >18 months of social isolation and the implementation of restrictions due to the COVID-19 pandemic in Chile.

We hypothesized the following points: (1) a higher BMI and waist-to-height ratio (WtHR) will be associated with lower scores in terms of executive function, and (2) children with lower performances on tests that measure physical fitness and lower physical activity levels will show lower executive function performance.

## 2. Materials and Methods

### 2.1. Design and Participants

This cross-sectional study included 90 boys and girls (male gender, *n* = 46; female gender, *n* = 44) from a school in the Las Condes commune, Santiago de Chile (Chile), in the fifth and sixth grade, aged between 10 and 12 years (M ± SD = 11.45 ± 0.68 years). The subjects’ socioeconomic level was identified as “high” considering the socioeconomic status index estimated for 2019 for their place of residence, according to Mena et al. [39]. The study protocol was approved by the ethics review board from the Scientific Ethics Committee of the University Andres Bello, Chile (no. 009/2021). The procedure was performed in accordance with the latest version of the Declaration of Helsinki (2013).

### 2.2. Measures

#### 2.2.1. Executive Functions 

The Neuropsychological Assessment for Executive Function in Children Battery (in Spanish (original language): Evaluación Neuropsicológica de las Funciones Ejecutivas en Niños-ENFEN) [40], whose application is indicated for Spanish-speaking school children between 6 and 12 years of age, was used. In this study, the assessment lasted approximately 30 to 40 min per participant and consisted of four tests that allow for the evaluation of the neuropsychological development of students in activities related to executive functions, yielding a direct score for each task, from which it is possible to obtain typical scores that are expressed on a scale of sten scores (M = 5.5, SD = 2). 

The first test is called “verbal fluency” and is composed of two parts: “phonological fluency” (F1) and “semantic fluency” (F2). Phonological fluency assesses the subject’s ability to say as many words beginning with “M” as possible in one minute, while the second part assesses their ability to say as many words belonging to the “animals” category as possible, also in one minute. Each part has a test rehearsal. The direct score corresponds to the number of correct words. The second test is the “trails” test and also consists of two parts: the “gray trail” (S1) and the “color trail” (S2). S1 consists of joining a series of numbers on a sheet of paper in descending order (20 to 1) as quickly as possible. S2 consists of joining a series of numbers in ascending order (1 to 21), alternating the color of the successive numbers (yellow and pink) as quickly as possible. Each of these parts has a test rehearsal as well. The time the student takes to complete each part must be recorded, and the direct score is obtained through calculation using a formula in which the number of correct answers, number of errors, and time spent are entered. The third test is called the “rings task” (A) and consists of 15 trials in which a set of rings (increasing with the number of tests) must be moved from an initial position into a final position, using specific material to replicate a drawing found in the stimulus notebook. This test also features a rehearsal. The time spent by the student on each trial is recorded, and the direct score is the sum of the times (in seconds) of all the trials. The fourth test is the “interference” (IN) test (inspired by the Stroop effect), in which students must name the color of the ink in which 39 words (color names) are printed and divided into three columns. The name of the color and the color of the ink in which each word is printed never matches. The student must name the color of the ink for each word. The total time taken to complete the test, number of successes, and number of errors are recorded, which are then incorporated into a formula to obtain the direct score of the test. This test has a rehearsal as well.

The ENFEN battery has been widely used for clinical and research purposes [41,42,43,44,45].

#### 2.2.2. Physical Activity

Participants’ physical activity was assessed using the Physical Activity Questionnaire for Older Children (PAQ-C), Spanish validated version [46], which was designed to be administered to primary school children, approximately aged between 8 and 14 years. This questionnaire assesses physical activity practice in the last 7 days through nine questions about its type and frequency. It also consists of a 10th item that is used to identify students who performed any unusual activity during the previous week, without receiving a score. Responses are scored on a five-point scale. 

#### 2.2.3. Anthropometric Parameters and Physical Fitness

Body height was measured without shoes, with a precision of 0.1 mm and a range of 0–2.50 m, using a portable stadiometer (Seca & Co. KG, Hamburg, Germany). Body weight (kg) was evaluated wearing a T-shirt, shorts, and no shoes, using a digital scale (TANITA^TM^, model 331, Tokyo, Japan). These anthropometric variables were measured according to the procedure described by Ross and Marfell-Jones [47]. The BMI was calculated as body mass divided by the square of the height in meters (kg/m^2^). In addition, waist circumference was measured (cm) over the rim of the iliac crest, through the umbilicus, using a flexible measuring tape, with an accuracy of 0.1 cm. The WtHR was obtained by dividing the waist circumference (WC) by the height. A WtHR of >0.5 is considered a cardiometabolic risk factors according to international standards [48].

To evaluate lower-body muscular power, the study subjects performed the standing long jump test (SLJ) [49] in which participants jump forward as far as possible, swinging their arms, bending their knees, and taking both legs off the floor at the same time. The distance that the subjects can jump was measured in centimeters (cm) from the starting line marked on the floor to the landing point at the back of the heel. To measure sprint performance, the subjects performed the 10 × 5 m agility shuttle run test [49] in which participants run as fast as possible between two cones that are placed 5 m apart from each other, performing a total of five cycles (50 m). The time taken by each participant to complete the five cycles is measured in seconds. Upper-body muscular strength was assessed using handgrip strength (HGS) with a hydraulic dynamometer (JAMAR Hydraulic Hand Dynamometer^®^ Model PC-5030 J1, Fred Sammons, Inc., Burr Ridge, IL, USA), with an accuracy of 0.1 lbf. In this test, participants had to hold the handgrip dynamometer with their dominant hand (with their arm hanging at the side of their body) and squeeze it as hard as possible for at least 2 s. The best of two attempts [49] was considered. The six-minute walk test (SMWT) is a submaximal exercise test, which was used to evaluate aerobic fitness [50,51,52]; participants were asked to walk as fast as possible, without running, in a 30-m-long corridor that was marked at each end. The distance traveled in meters after 6 min was recorded. 

### 2.3. Procedure

In the first instance, the study characteristics and scope were discussed with the governing body and teachers of the school. Once the school’s authorization was obtained, students’ parents or legal guardians were informed by means of written communication, and informed consent was obtained. Next, the students whose participation was authorized were informed about the characteristics of the study, and their informed assent was requested. 

Once informed consents and assents were obtained, measurements were performed using the reported instruments over two sessions (30 August and 3 September 2021) within the school day. In the first session, anthropometric parameters (weight, size, BMI, WC, and WtHR) were evaluated by the school nurse, who has more than 10 years of professional experience and was trained by the research team to perform these measurements, and the tests to measure students’ physical fitness status (SMWT, SLJ test, 10 × 5 m agility shuttle run test, and HGS assessment) were performed by the school’s physical education teachers team, who also received previous training. In the second session, the ENFEN battery was used to evaluate executive function and the PAQ-C questionnaire was used to assess the physical activity level. These last evaluations were performed by a team of speech therapists who were specially trained in terms of the direct and individual application of these instruments. For each participant, assessments were performed in a separate room that was free of distractions and always in the same order, ensuring that the application conditions were the same for all students.

### 2.4. Statistical Analysis

Statistical analysis was performed using STATA v15.0 software (StataCorp, College Station, TX, USA). Normal distribution was tested using the Shapiro–Wilk test. As all variables were continuous, values have been presented as mean and SD. Differences between the sexes were determined using Student’s t-test. To determine the association between anthropometric parameters and fitness, a multivariate linear regression analysis was performed, including 95% confident interval (CI) values for correlations. Values of *p* < 0.05 were considered statistically significant. The correlation values (r) were interpreted according to Akoglu [53]. 

## 3. Results

### 3.1. Anthropometric Parameters, Physical Fitness, and Executive Functions in Children According to Sex

Table 1 shows the age, anthropometric characteristics, physical fitness status, and executive functioning tasks scores obtained with the ENFEN battery in children according to sex. In terms of anthropometric parameters, boys had higher WtHR (*p* < 0.001) and lower physical activity levels (*p* = 0.049) and HGS (*p* = 0.009) than girls. The ENFEN-related scores were similar for both sexes.

### 3.2. Anthropometric Parameters and Executive Functions

Table 2 shows the linear relationship between the anthropometric parameters and the ENFEN tasks. The increase in the BMI showed a negative correlation with F1 (r = −0.50, *p* ≤ 0.001) and S1 (r = –0.53, *p* < 0.001), whereas the increased WtHR showed a negative relation with F2 (r = −0.37, *p* = 0.002) and S1 (r = −0.42, *p* < 0.001). 

### 3.3. Physical Fitness and Executive Functions

Table 3 shows the linear relationship between physical fitness levels and ENFEN tasks. In particular, the SLJ test scores were positively correlated with F1 (r = 0.24, *p* = 0.02) and S1 (r = 0.21, *p* = 0.04) tasks, and the HGS was positively correlated with F2 (r = 0.23, *p* = 0.02), S1 (r = 0.26, *p* = 0.01), and IN (r = 0.20, *p* = 0.05). Furthermore, the 10 × 5 m sprint was negatively correlated with poor executive functioning (r = −0.54 to −0.26; *p* = 0.01 to <0.001).

### 3.4. Effect of Anthropometric and Fitness Parameters on Executive Functions

Table 4 shows the association between the ENFEN tasks and anthropometric and physical fitness parameters in a model adjusted by age and sex. For the sten for F1, F2, S1, S2, and IN parameters, there was an inverse association between weight, BMI, WC, and WtHR. The anthropometric parameters were found to be associated with poor executive functioning (i.e., higher values of anthropometric parameters were associated with a poorer executive functioning). Likewise, in terms of physical fitness, an inverse association was found between the 10 × 5 m sprint and sten F1, F2, S1, A, and IN. In particular, A showed only an inverse effect on the 10 × 5 m sprint (β = –0.58 (–0.91 to –0.25), *p* = 0.001). On the other hand, a positive effect was found between executive functioning and the physical activity score, principally with the F2 (β = 0.11 (0.03 to 0.19), *p* = 0.005) and IN sten (β = 0.13 (0.05 to 0.22), *p* = 0.002).

## 4. Discussion

This study examined the possible associations between anthropometric parameters, physical activity and fitness, and executive functions among primary school children at the time of their return to school in person after the restrictions imposed due to the COVID-19 pandemic in Chile were lifted.

The results obtained indicated that the increase in the selected anthropometric parameters (weight, BMI, WC, and WtHR) was associated with a lower performance in all executive function and attention tasks. Although the tasks used to assess executive function involve complex demands that make it difficult to identify the unique contribution of a single dimension of executive functions [54,55], the results allow us to relate the increase in these anthropometric parameters to a lower performance in the following: (1) inhibitory control, by means of the IN task, which is based on the Stroop effect that assumes prolonged response time and greater propensity to make errors in the face of contradictory data [54,56,57]; (2) cognitive flexibility, by means of the F1 and F2 tasks (together, verbal fluency test) [54,58,59] and the trails test, which resembles the well-known Trail Making Test [60,61]; and (3) planning, via the A task, which is a modification of the Hanoi Towers Test [45,54,62]. Verbal fluency tasks have also been considered as an indirect measure of working memory [45,62] and the S1 task (part 1 of the trails test) is closely associated with attention [60].

Our findings are consistent with those of Ronan et al. [38], as in their study, executive function was found to be significantly associated with physical activity, BMI, WC, and WtHR in children. This relationship could be partially explained by increased BMI-associated reduction in prefrontal cortical thickness. The prefrontal cortex is involved in decision-making, inhibition, working memory, and cognitive flexibility; a reduction in its thickness could be considered a risk factor for the developing brain [38,63]. Along the same lines, Alarcón et al. [64] reported that BMI was inversely related to both verbal and spatial working memory accuracy and to the white matter microstructure of association fibers that connect brain regions associated with working memory ability in a sample of adolescents. On the other hand, Li et al. [18] conducted a longitudinal study to estimate the associations between early-life weight status and cognition in a sample of typically developing children. They concluded that early-life weight status was negatively associated with children’s perceptual reasoning and working memory. Other studies that focused on the relationship of higher BMI and excessive body weight (being overweight/obese) with executive functions among children show a reciprocal influence between these variables. However, despite the lack of sufficient evidence to establish the directionality of this relationship, they all describe deficits in different executive function dimensions and other cognitive aspects relevant to daily functioning, such as attention and processing speed, that are associated with these conditions [63,65,66,67,68,69]. 

There is lack of information regarding the relationship of WC and WtHR with executive function among children; however, our results agree with the results of a study by Huang et al. [70], who showed that BMI and WC were negatively associated with inhibitory control, regardless of aerobic fitness, in a large sample of adolescents. Kamijo et al. [71] also support the relationship between BMI and fat mass as markers of adiposity with lower performance in terms of inhibitory control and academic performance in preadolescents. Flores et al. [72] reported similar results, providing background information that associates cognitive performance (attention, memory, and executive functions) among children and adolescents with strong predictors of cardiovascular disease, including WtHR and stress hormones, indicating that cognitive test scores decrease as the number of involved cardiovascular risk factors increases. Poorer planning performance has also been associated with greater WC among children with sedentary lifestyles and typical development [73].

Physical fitness and activity are important markers of both physical and mental health that are associated with cognitive development during childhood and adolescence, especially with regard to executive functions [5,6,7,8]. Physical activity refers to any body movement produced by skeletal muscles that leads to energy expenditure, whereas physical fitness refers to a set of physical attributes or skills that can be measured using specific tests [6,74]. Currently, the human motor ability is understood in a multidimensional way in which there are basic motor skills (endurance, strength, speed, coordination, and flexibility) that are described as being more energy demanding (such as endurance) or more information oriented (such as coordination) or that share both components (such as muscle power) [75,76]. This model allows us to understand that motor skills are associated with cognitive skills to different degrees and especially with executive function, which acts as a mediator between physical-activity-related outcomes and learning among children [77]. 

The results obtained in this study regarding the relationship of physical activity and fitness with executive function tasks show the positive effect of physical activity on working memory, cognitive flexibility, inhibition, and attention. The physical test that showed a significant association with the greatest number of cognitive tasks was the 10 × 5 m sprint as it was associated with all of the tasks, except for the S2 task (which is mainly associated with cognitive flexibility) in which the results were not significant. This means that improving sprint performance also improves performance in working memory, cognitive flexibility, inhibition, planning, and attention. The S2 task showed no significant association with fitness parameters, and the SMWT showed no significant relationship with executive functioning tasks. The analysis of the relationship between muscular strength and executive function shows that an increase in lower-body muscular power (SLJ test) is associated with significant improvements in working memory skills, cognitive flexibility, and attention, whereas a higher performance in upper-body muscular strength (HGS) is significantly associated with better performance in working memory, cognitive flexibility, attention, inhibition, and planning. 

Previous studies that have analyzed the association between physical activity and executive function among children are in agreement that a higher level of physical activity is positively associated with a greater development of executive function among children [10,78]. Our results also agree with those of a study by Mora et al. [15], which analyzed the relationship of physical fitness and activity with executive function among children who were overweight and obese, finding a significant positive association between HGS and planning ability. Other studies do not allow us to identify the specific relationship between muscle strength and executive functions, as they combined muscle strength tests with other physical tests to obtain a global measure of physical fitness [17,79]. Schmidt et al. [77], on the other hand, did not find a significant direct effect of the applied measure for strength (SLJ) on executive functions (for working memory, inhibition, and cognitive flexibility tasks) among children. Future research will be necessary to corroborate these results.

Veraksa et al. [6] analyzed the potential association between physical fitness and executive functions among preschoolers (5 and 6 year olds), and their results indicated that higher levels of physical fitness also increased performance in two of the three main executive function dimensions: working memory and inhibition. These authors found no differences with regard to cognitive flexibility as a function of physical fitness; however, the motor tasks that accounted the most for the differences in inhibitory control and working memory (both verbal and visual–spatial) were shuttle running (shuttle run 4 × 5 m) and throwing, which required a higher level of motor coordination compared to the other tasks. Although the ages differ from those included in our study, the results of both studies coincide with the two dimensions of executive function that showed a linear relationship with the physical fitness tests (inhibitory control and working memory), although our study also found that there was a significant relationship with regard to planning (A task), attention (S1 task), and cognitive flexibility (verbal fluency). In both studies, the shuttle running task was most often associated with executive functions. Mora et al. [15] also reported a positive association between speed–agility (4 × 10 m shuttle run test), cognitive flexibility and inhibition. These findings are in accordance with previous studies in which physical tasks involving motor coordination were mostly associated with cognitive skills and, in particular, with executive functions [17,77,80].

Much of the currently available evidence suggests that cardiorespiratory fitness plays an important role in the development of executive functions during childhood and adolescence. However, some studies have only showed relationships with some of the dimensions analyzed [6,70,81,82,83,84]. Meijer et al. [85] evaluated the association between cardiovascular fitness (i.e., the 20 m shuttle run test) and a set of neurocognitive function measures (including executive function measures in terms of working memory, motor inhibition, and inhibition control) in a large sample of primary school children, finding significant results only for visuospatial working memory in the executive function domain. A second example is the study conducted by Schmidt et al. [77], who did not identify a significant direct effect between the results of an aerobic fitness task (a multistage 20 m shuttle run test) and executive function for any of its three main dimensions in a sample of children aged 10 to 12 years. Some studies even suggest that the relationship between aerobic fitness and executive functions remains debatable as the results provided by research are inconsistent [86,87]. This reflects the fact that searching for relationships between these variables requires an exhaustive and precise analysis of the influence by modulating factors that affect data collection, irrespective of whether they are biological, psychological, or others [88]. In our case, the results obtained from the SMWT showed no significant relationship with executive functioning tasks, which could be influenced to some extent by the characteristics of this test as it is described as a submaximal exercise test. However, tests were selected based on the explicit request of the school, who did not want to subject the children to high-demand physical tasks (such as the 20 m shuttle run test) that could generate discomfort due to, for example, the need to wear a facemask at all times and the fact that the students had not been exposed to high physical demands for a long period of time. The significant reduction in physical activity levels and increase in sedentary behaviors (such as increased screen time and sitting time) has been documented nationally and internationally during the COVID-19 pandemic period [35,89,90,91]. 

This study provides novel background information that highlights the importance of paying attention to children without associated morbidities. It seeks preventive strategies to enhance their performance during the crucial stage of development, where school children move from late childhood to adolescence, facing academic demands that require an optimal cognitive level. In addition, the specific timing of the data collection (return to the in-person school context after an extensive period of restrictions on mobility and free movement during the COVID-19 pandemic) increases the importance of further analyzing these variables related to health (physical and mental), physical activity, and the physical condition of school children both in Chile and globally, in view of the evidence indicating pandemic-related damage [20,30,34,35,92,93]. Some successful intervention experiences exemplify the benefits of addressing this problem early, showing a positive impact on BMI, physical activity level, and the academic performance of elementary school children [94,95]. Studies have also shown that a combination of moderate to vigorous physical exercise and cognitively demanding activities, which applies especially to the practice of sports, will have a greater effect on executive functioning [5,7,96,97,98,99,100]. 

One of this study’s advantages was that it analyzed the associations between anthropometric parameters, physical activity and fitness, and executive functions, in detail by incorporating tests that are not commonly used to assess executive function among children, such as the SLJ test and HGS, to account for muscle power and strength. One limitation of the study is its cross-sectional design, which, to some extent, restricts the scope of the conclusions. In addition, the sample was obtained from a private school in an urban area, which limits the generalization of the results to all socioeconomic strata in which children develop. Future research should include a larger sample size to help identify more consistent associations between variables. Finally, longitudinal analyses in terms of sports, physical fitness, and executive functions should be performed, given the importance of incorporating variables that allow us to identify the impact of ecological interventions that are useful in the educational context. This could favor the generation of strategies that enhance children’s cognitive development and academic success at the school age.

## 5. Conclusions

Anthropometric parameters and physical activity and fitness are associated with executive functions and attention in the school-age population. 

For a healthy brain, current findings support the need for interventions that favor healthy anthropometric indexes, increased levels of physical activity, and improved physical fitness among the school-age population. 

## Figures and Tables

**Table 1:** Age and scores of anthropometric parameters, physical fitness, and ENFEN tasks secured by children according to sex.

Children’s Characteristics	Total (*n* = 90)	Girls (*n* = 44)	Boys (*n* = 46)	*p*-Value
Age	11.45 ± 0.68	11.43 ± 0.71	11.46 ± 0.66	0.88
Anthropometrics				
Weight (kg)	43.9 ± 4.87	43.3 ± 9.83	43.6 ± 7.78	0.72
Height (m)	1.5 ± 0.06	1.53 ± 0.04	1.47 ± 0.07	<0.001
WC (cm)	70.7 ± 7.86	68.9 ± 4.04	72.3 ± 10.06	0.042
WtHR (WC/height)	0.47 ± 0.05	0.45 ± 0.03	0.48 ± 0.06	<0.001
BMI (kg/m^2^)	19.26 ± 3.1	18.8 ± 2.2	19.71 ± 3.74	0.15
Physical fitness				
PAQ-C	2.76 ± 0.73	2.91 ± 0.62	2.6 ± 0.80	0.049
SMWT (m)	365.6 ± 68.9	347.2 ± 22.5	383.2 ± 90.9	0.012
10 × 5 m sprint (s)	22.6 ± 2.4	22.0 ± 1.69	23.2 ± 2.84	0.021
SLJ (cm)	143.7 ± 18.2	148.5 ± 17.8	139.2 ± 17.6	0.014
HGS (kg)	17.2 ± 5.3	18.6 ± 5.9	15.7 ± 4.2	0.009
ENFEN tasks				
Phonological fluency (F1)	11.46 ± 4.15	11.61 ± 3.98	11.32 ± 4.34	0.74
Sten F1	4.8 ± 2.5	4.8 ± 2.3	4.8 ± 2.7	0.97
Semantic fluency (F2)	18.85 ± 4.26	18.47 ± 4.61	19.2 ± 3.92	0.41
Sten F2	6.1 ± 1.89	5.8 ± 1.97	6.47 ± 1.77	0.11
Gray trails (S1)	32.2 ± 11.2	34.2 ± 11.74	30.28 ± 10.4	0.09
Sten S1	6.3 ± 2.2	6.7 ± 2.1	6.0 ± 2.3	0.15
Color trails (S2)	16.4 ± 5.55	16.8 ± 5.6	15.99 ± 5.5	0.48
Sten S2	4.8 ± 1.9	5.0 ± 2.0	4.7 ± 2.0	0.47
Rings task (A)	183.1 ± 28.8	179.8 ± 26.5	186.2 ± 30.8	0.29
Sten A	4.1 ± 1.43	4.1 ± 1.28	4.1 ± 1.57	0.95
Interference (IN)	81.1 ± 18.7	83.1 ± 17.3	79.1 ± 20.0	0.31
Sten IN	5.2 ± 1.63	5.2 ± 1.5	5.2 ± 1.77	0.98

The data shown represent the mean ± standard deviation. ENFEN = Neuropsychological Assessment for Executive Function in Children Battery; BMI = body mass index; WC = waist circumference; WtHR = waist-to-height ratio; PAQ-C = Physical Activity Questionnaire for Older Children; SMWT = six-minute walk test; SLJ = standing long jump test; HGS = handgrip strength.

**Table 2:** Linear relationship between anthropometric parameters and ENFEN tasks performed by Chilean children.

Children Characteristics	Weight	BMI	WC	WtHR
ENFEN tasks				
Phonological fluency score (F1)	–0.37 (<0.001)	–0.50 (<0.001)	–0.35 (<0.001)	–0.35 (<0.001)
Sten F1	–0.42 (<0.001)	–0.49 (<0.001)	–0.37 (<0.001)	–0.30 (0.003)
Semantic fluency score (F2)	–0.30 (0.004)	–0.43 (<0.001)	–0.40 (<0.001)	–0.37 (0.002)
Sten F2	–0.32 (0.001)	–0.39 (<0.001)	–0.38 (<0.001)	–0.28 (0.006)
Gray trails score (S1)	–0.30 (0.003)	–0.53 (<0.001)	–0.34 (0.001)	–0.42 (<0.001)
Sten S1	–0.36 (<0.001)	–0.55 (<0.001)	–0.36 (<0.001)	–0.41 (<0.001)
Color trails score (S2)	–0.11 (0.26)	–0.22 (0.030)	–0.06 (0.53)	–0.08 (0.45)
Sten S2	–0.12 (0.24)	–0.22 (0.031)	–0.05 (0.60)	–0.06 (0.53)
Rings task score (A)	–0.02 (0.79)	0.12 (0.24)	0.15 (0.13)	0.22 (0.02)
Sten A	–0.06 (0.54)	–0.10 (0.34)	–0.13 (0.19)	–0.08 (0.41)
Interference score (IN)	–0.10 (0.34)	–0.16 (0.11)	–0.12 (0.24)	–0.17 (0.08)
Sten IN	–0.18 (0.08)	–0.14 (0.18)	–0.05 (0.59)	0.004 (0.96)

The data shown represent the correlation coefficient r (*p*-value). Values of *p* < 0.05 were considered statistically significant. ENFEN = Neuropsychological Assessment for Executive Function in Children Battery; BMI = body mass index; WC = waist circumference; WtHR = waist-to-height ratio.

**Table 3:** Linear relationship between fitness parameters and ENFEN tasks performed by Chilean children.

Children Characteristics	PAQ-C	SMWT	SLJ	10 × 5 m Sprint	HGS
ENFEN tasks					
Phonological fluency score (F1)	0.19 (0.06)	–0.06 (0.51)	0.24 (0.02)	–0.33 (0.001)	0.17 (0.10)
Sten F1	0.21 (0.04)	–0.08 (0.43)	0.19 (0.06)	–0.32 (0.001)	0.04 (0.66)
Semantic fluency score (F2)	0.19 (0.04)	0.05 (0.64)	0.17 (0.09)	–0.48 (<0.001)	0.23 (0.02)
Sten F2	0.19 (0.06)	0.02 (0.82)	0.13 (0.21)	–0.46 (<0.001)	0.13 (0.20)
Gray trails score (S1)	0.18 (0.08)	0.08 (0.42)	0.21 (0.04)	–0.52 (<0.001)	0.26 (0.01)
Sten S1	0.23 (0.02)	0.04 (0.65)	0.21 (0.03)	–0.54 (<0.001)	0.21 (0.04)
Color trails score (S2)	0.08 (0.39)	–0.08 (0.41)	–0.02 (0.83)	–0.07 (0.45)	0.13 (0.19)
Sten S2	0.09 (0.35)	–0.13 (0.20)	–0.006 (0.99)	–0.12 (0.23)	0.08 (0.40)
Rings task score (A)	0.09 (0.39)	–0.05 (0.61)	–0.14 (0.17)	0.40 (<0.001)	–0.31 (0.002)
Sten A	–0.000 (0.99)	–0.09 (0.35)	0.10 (0.33)	–0.40 (<0.001)	0.10 (0.32)
Interference score (IN)	0.07 (0.51)	–0.02 (0.83)	0.14 (0.16)	–0.29 (0.005)	0.20 (0.04)
Sten IN	0.22 (0.03)	–0.18 (0.08)	0.14 (0.15)	–0.26 (0.01)	0.02 (0.80)

The data shown represent the correlation coefficient r (*p*-value). Values of *p* < 0.05 were considered statistically significant. ENFEN = Neuropsychological Assessment for Executive Function in Children Battery; PAQ-C = Physical Activity Questionnaire for Older Children; SMWT = six-minute walk test; SLJ = standing long jump test; HGS = handgrip strength.

**Table 4:** Association between anthropometric and fitness parameters with ENFEN parameters among Chilean children.

Sten Scores	F1	F2	S1	S2	A	IN
	β (95% CI) *p*-Value	β (95% CI) *p*-Value	β (95% CI) *p*-Value	β (95% CI) *p*-Value	β (95% CI) *p*-Value	β (95% CI) *p*-Value
Anthropometric parameters						
Weight (kg)	–1.41 (–2.0 to –0.81), *p* < 0.001	–1.2 (–2.1 to –0.33), *p* = 0.007	–1.83 (–2.53 to –1.14), *p* < 0.001	–1.25 (–2.1 to –0.4), *p* = 0.004	–0.58 (–1.74 to 0.57), *p* = 0.32	–1.2 (–2.2 to –0.2), *p* = 0.018
WC (cm)	–1.32 (–1.9 to –0.73), *p* < 0.001	–1.44 (–2.3 to –0.61), *p* = 0.001	–1.6 (–2.3 to –0.89), *p* < 0.001	–1.1 (–1.9 to –0.24), *p* = 0.012	–0.68 (–1.83 to 0.46), *p* = 0.23	–1.4 (–2.3 to –0.43), *p* = 0.005
WHtR (WC/height)	–0.007 (–0.01 to –0.003), *p* < 0.001	–0.009 (–0.01 to –0.003), *p* = 0.001	–0.009 (–0.01 to –0.006), *p* < 0.001	–0.007 (–0.01 to –0.001), *p* = 0.012	–0.003 (–0.01 to 0.004), *p* = 0.4	–0.01 (–0.01 to –0.004), *p* < 0.001
BMI (kg/m^2^)	–0.55 (–0.78 to –0.32), *p* < 0.001	–0.51 (–0.84 to –0.18), *p* = 0.003	–0.85 (–1.1 to –0.6), *p* < 0.001	–0.55 (–0.88 to –0.23), *p* = 0.001	–0.17 (–0.63 to 0.27), *p* = 0.43	–0.64 (–1.0 to –0.3), *p* = 0.001
Physical fitness						
PAQ-C	0.07 (0.02 to 0.13), *p* = 0.009	0.11 (0.03 to 0.19), *p* = 0.005	0.09 (0.02 to 0.16), *p* = 0.006	0.06 (–0.01 to 0.14), *p* = 0.08	–0.004 (–0.11 to 0.1), *p* = 0.93	0.13 (0.05 to 0.22), *p* = 0.002
SMWT (m)	1.67 (–3.9 to 7.3), *p* = 0.55	1.17 (–6.43 to 8.8), *p* = 0.76	–4.64 (–11.3 to 2.0), *p* = 0.17	–10.3 (–17.5 to –3.1), *p* = 0.006	–4.6 (–14.4 to 5.2), *p* = 0.35	–6.4 (–14.9 to 2.1), *p* = 0.14
SLJT (cm)	1.14 (–0.29 to 2.6), *p* = 0.11	1.22 (–0.73 to 3.18), *p* = 0.21	1.37 (–0.35 to 3.1), *p* = 0.11	–1.38 (–3.3 to 0.54), *p* = 0.15	0.92 (–1.6 to 3.5), *p* = 0.47	1.23 (–0.98 to 3.46), *p* = 0.27
10 × 5 m sprint (s)	–0.34 (–0.53 to –0.15), *p* < 0.001	–0.59 (–0.83 to –0.35), *p* < 0.001	–0.58 (–0.8 to –0.38), *p* < 0.001	–0.01 (–0.3 to 0.25), *p* = 0.92	–0.58 (–0.91 to –0.25), *p* = 0.001	–0.41 (–0.7 to –0.12), *p* = 0.006
HGS (kg)	–0.11 (–0.51 to 0.3), *p* = 0.59	0.29 (–0.25 to 0.83), *p* = 0.29	0.22 (–0.26 to 0.70), *p* = 0.37	–0.07 (–0.61 to 0.47), *p* = 0.8	0.32 (–0.38 to 1.0), *p* = 0.37	0.10 (–0.52 to 0.72), *p* = 0.74

Data shown represent the β coefficient (95% CI) adjusted by age and sex. ENFEN = Neuropsychological Assessment for Executive Function in Children Battery; BMI = body mass index; WC = waist circumference; WtHR = waist-to-height ratio; SLJ = standing long jump test; PAQ-C = Physical Activity Questionnaire for older Children; SMWT = six-minute walk test; HGS = handgrip strength.

## Data Availability

The datasets generated and analyzed for this study can be obtained from the corresponding author.
